# Cognitive function after transcatheter aortic valve implantation: the forgotten child

**DOI:** 10.1007/s12471-023-01821-z

**Published:** 2023-10-19

**Authors:** Hugo M. Aarts, Michiel Voskuil

**Affiliations:** Department of Cardiology, University Medical Centre Utrecht, Utrecht, The Netherlands

Transcatheter aortic valve implantation (TAVI) has become routine treatment in patients with aortic valve stenosis and an increased surgical risk. Although the risk of periprocedural complications has decreased dramatically over the last decade, ischaemic stroke still remains a feared and detrimental complication in patients undergoing TAVI, with an estimated incidence between 2 and 4% [1]. In addition to the occurrence of these ischaemic cerebrovascular events, the number of new cerebral lesions on magnetic resonance imaging after TAVI has been shown to be associated with the occurrence of delirium following TAVI [2]. Moreover, changes in neurocognitive functioning frequently occur after both surgical aortic valve replacement and TAVI [3]. These neurological complications are primarily caused by dislodged material (e.g. pieces of calcium, tissue or thrombus) that embolise towards the brain [4].

In recent years, different catheters have been developed to minimise the risk of neurological complications, such as the filter-based catheter to catch debris in the head and neck vessels and the deflection filter made of fine nitinol that covers all 3 vessels. Observational studies have shown a reduction in the number and volume of cerebral lesions with the use of these embolic protection devices [5]. Last year, the results of the first randomised study looking at hard clinical endpoints were published. The PROTECTED TAVR study, including 3000 patients, confirmed the safety of the filtered-based cerebral protection device but could not demonstrate a reduction in the incidence of stroke [6]. However, a subgroup analysis did show that the use of this filter device reduced the risk of disabling stroke [6]. Another large trial that will randomise 7730 patients to TAVI either with or without a cerebral protection device, the PROTECT-TAVI trial, is currently ongoing [7]. Therefore, a definite answer to whether cerebral protection with the current-generation catheters can prevent stroke in patients undergoing TAVI still has to be awaited.

As mentioned above, ischaemic cerebral lesions due to embolised particles can lead to impaired cognitive functioning. In contrast, TAVI may also have positive cognitive effects as it leads to increased cardiac output and cerebral blood flow [8]. Interestingly, the exact effects of TAVI on cognitive functioning have not been investigated systematically. This has led to the design of the CAPITA (CArdiac outPut, cerebral blood flow and cognition In patients with severe aortic valve stenosis undergoing Transcatheter Aortic valve implantation) study, which is described by Van Nieuwkerk and coworkers in the current issue of the *Netherlands Heart Journal* [9]. It will be the first large-scale study aimed at prospectively and systematically assessing cognition and cerebral blood flow (CBF) in patients undergoing TAVI. The hypothesis of the study is that improved cardiac output after TAVI results in increased CBF with subsequent positive effects on cognitive functions. Therefore, brain magnetic resonance imaging (measuring CBF) and extensive neuropsychological testing will be performed before and after TAVI (at 3 months and 1 year). The investigators aimed to include 152 patients to prove their hypothesis. The last patient was included in October 2022, and the first study results are expected by the end of 2023. The results of the CAPITA study will teach us about the potential cognitive benefit of TAVI due to improved haemodynamics and enhance our understanding of the relationship between cardiac function, cerebral blood flow and cognitive functioning.

Further expansion of indications for TAVI towards lower-risk and younger patients is expected in the coming years. Therefore, continuous improvements in the TAVI procedure, patient selection and optimal treatment choice are crucial. Whereas trials investigating cerebral protection devices will help to improve clinical outcomes of patients undergoing TAVI, the CAPITA study will lead to better understanding of the heart-brain axis with easier identification of the patient that will benefit the most from TAVI.
